# Adipocyte-Specific Expression of PGC1α Promotes Adipocyte Browning and Alleviates Obesity-Induced Metabolic Dysfunction in an HO-1-Dependent Fashion

**DOI:** 10.3390/antiox11061147

**Published:** 2022-06-10

**Authors:** Shin-Hsueh Shen, Shailendra P. Singh, Marco Raffaele, Maayan Waldman, Edith Hochhauser, Juancarlos Ospino, Michael Arad, Stephen J. Peterson

**Affiliations:** 1 Department of Pharmacology, New York Medical College, Valhalla, NY 10595, USA; hsshen@mail.ndmctsgh.edu.tw (S.-H.S.); spbiotech2004@gmail.com (S.P.S.); 2 Department and Institute of Pharmacology, National Defense Medical Center, Taipei 11490, Taiwan; 3 Department of Drug Science, University of Catania, 95125 Catania, Italy; marco.raffaele@hotmail.com; 4 Cardiac Research Laboratory, Felsenstein Medical Research Institute Petah-Tikva, Sackler Faculty of Medicine, Tel Aviv University, Tel Aviv 6997801, Israel; maayanw@gmail.com (M.W.); hochhaus@tauex.tau.ac.il (E.H.); 5 Leviev Heart Center, Sheba Medical Center, Tel Hashomer and Sackler School of Medicine, Tel Aviv University, Tel Aviv 6997801, Israel; michael.arad@sheba.health.gov.il; 6 Department of Medicine, New York-Presbyterian Brooklyn Methodist Hospital, Brooklyn, NY 11215, USA; juancarlosospino@gmail.com; 7 Department of Medicine, Weill Cornell Medicine, New York, NY 10065, USA

**Keywords:** brown fat, PGC-1α, mitochondria, inflammation, type 2 diabetes, obesity

## Abstract

Recent studies suggest that PGC1-α plays a crucial role in mitochondrial and vascular function, yet the physiological significance of PGC1α and HO expression in adipose tissues in the context of obesity-linked vascular dysfunction remains unclear. We studied three groups of six-week-old C57BL/6J male mice: (1) mice fed a normal chow diet; (2) mice fed a high-fat diet (H.F.D.) for 28 weeks, and (3) mice fed a high-fat diet (H.F.D.) for 28 weeks, treated with adipose-specific overexpression of PGC-1α (transgenic-adipocyte-PGC-1α) at week 20, and continued on H.F.D. for weeks 20–28. R.N.A. arrays examined 88 genes involved in adipocyte proliferation and maturation. Blood pressure, tissue fibrosis, fasting glucose, and oxygen consumption were measured, as well as liver steatosis, and the expression levels of metabolic and mitochondrial markers. Obese mice exhibited a marked reduction of PGC1α and developed adipocyte hypertrophy, fibrosis, hepatic steatosis, and decreased mitochondrial respiration. Mice with adipose-specific overexpression of PGC1-α exhibited improvement in HO-1, mitochondrial biogenesis and respiration, with a decrease in fasting glucose, reduced blood pressure and fibrosis, and increased oxygen consumption. PGC-1α led to the upregulated expression of processes associated with the browning of fat tissue, including UCP1, FGF21, and pAMPK signaling, with a reduction in inflammatory adipokines, NOV/CCN3 expression, and TGFβ. These changes required HO-1 expression. The R.N.A. array analysis identified subgroups of genes positively correlated with contributions to the browning of adipose tissue, all dependent on HO-1. Our observations reveal a positive impact of adipose-PGC1-α on distal organ systems, with beneficial effects on HO-1 levels, reversing obesity-linked cardiometabolic disturbances.

## 1. Introduction

Obesity is associated with developing severe metabolic disorders, including type 2 diabetes, metabolic syndrome, and fatty liver disease. Adiposity poses a significant risk for developing complications of obesity that include cardiometabolic dysfunction [1,2]. Approximately 171 million individuals have diabetes, a number expected to rise to 366 million by 2030 [3]. Adiposity is the precursor of hypertension fatty liver and metabolic syndrome, which contribute to the pathogenesis of heart failure (H.F.). Epidemiological studies suggest that more than 60% of the risk for primary hypertension may be attributed to obesity. In addition, long-standing hypertension is a major risk factor in the development of chronic kidney disease and H.F. [4,5,6]. Obesity leads to functional and structural metabolic changes in several organ systems, including liver, skeletal muscle and the brain. Obesity is a well-described risk factor for neurodegenerative diseases, such as Alzheimer’s, with oxidative stress and mitochondrial dysfunction contributing to the inflammatory cascade [6]. In the liver, obesity is the preamble to nonalcoholic fatty liver disease (NAFLD) and its complications. Indeed, the incidence of NAFLD and obesity are closely related [7,8,9].

Importantly, obesity leads to the accumulation of peripheral fat (adipose tissue) around the heart, liver, kidney, and other organs, priming insulin resistance and associated metabolic disturbances [10]. Peripheral and visceral adiposity are a major cause for the generation of reactive oxygen species (R.O.S.) and chronic inflammation, both being linked to the metabolic syndrome [11]. These factors promote the downregulation of the expression of peroxisome proliferator-activated receptor gamma coactivator 1-alpha (PGC-1α), which correlates with metabolic dysfunction and related pathophysiologic conditions. Reduced levels of PGC-1α are associated with reduced anti-oxidative properties and insulin resistance, critical features of obesity [12,13]. Human and murine studies have shown that lacking PGC-1α results in multi-organ challenges that are due to increased levels of reactive oxygen species (R.O.S.) [14,15]. Metabolic dysfunction plays a substantial role in the inflammation of adipocytes, resulting in a condition called adiposopathy [16,17].

The critical role of PGC-1α involves the master regulation and control of cellular energy metabolism that affects many tissues. PGC-1α expression stimulates mitochondrial biogenesis and the expression of antioxidant genes, heme oxygenase 1 (HO-1), and is increased in tissues and organs with high-energy metabolic loads, e.g., adipose tissue, cardiac, and skeletal muscle [18,19]. In adipose tissues, PGC-1α levels are regulated by stimuli that include physical activity and fasting in hepatic tissues [10,20]. Following activation, PGC-1α induces a transcription that initiates the biogenesis of mitochondrial and oxidative phosphorylation, resulting in tissue-specific gene remodeling. In contrast, the downregulation of PGC-1α expression is associated with metabolic dysfunction, inflammation, and altered redox control [4]. PGC-1α represents an attractive target with potential therapeutic benefits in obesity and associated cardiometabolic diseases [21,22].

We examine the impact of the adipocyte-specific expression of PGC-1α in a mouse model of obesity. We explore the effects on reprogramming white fat to brown-like fat and its repercussions in vascular and hepatic integrity. Our results represent the first evidence to date demonstrating that the selective overexpression of PGC-1α in adipose tissues has a long-lasting effect, preventing obesity-mediated inflammation and insulin resistance in obese mice in an HO-1-dependent manner.

## 2. Materials and Methods

### 2.1. Animal Experimentation and Generation of Transgenic Mice with Adipocyte-Specific Expression of PGC1α

Animal experiments were performed according to procedures approved by the Institutional Animal Care and Use Committee of New York Medical College. Six-week-old male C57BL/6J background mice were purchased from Jackson Labs (Bar Harbor, ME). The study involved three groups of animals (5 mice per group): (1) Lean, (2) H.F.D. control (28 weeks), and (3) adipose-specific PGC-1α transgenic mice on a H.F.D. for 28 weeks (20 weeks on a H.F.D. and then the transgene of PGC-1α followed by 8 more weeks of a H.F.D.). Lean mice were fed ad libitum with a normal chow diet (11% fat, 62% carbohydrate, 27.0% protein, total calories: 12.6 KJ/g). The high-fat diet (HFD) consisted of 58% fat (from lard), 25.6% carbohydrate, 16.4% protein, and total calories: 23.4 KJ/g (Bio-SERV, Frenchtown, NJ, USA) [23,24]. A lentiviral-mediated vector carrying PGC-1α (50 µL, 1 × 109 T.U./mL in saline solution was used to generate PGC1-α-transgenic mice after 20-weeks on a H.F.D. for an additional 8 weeks of H.F.D. after transfection. Mice were given bolus injections into the retro-orbital vein of the lentiviral constructs carrying PGC1-α under the control of the adiponectin promoter (Transgenic-Adipo-PGC1-α) to specifically target epididymal adipose tissue. The control H.F.D. mice were similarly injected with a transgenic-adipo-GFP (green fluorescent protein) vector. The control animals (group 1) were injected with mock virus (placebo) [17,25,26].

### 2.2. Generation of Lentiviral-Mediated PGC1-α Overexpression or Silencing in Adipocytes

3T3-L1 mouse pre-adipocytes were purchased from the ATCC (ATCC, Manassas, VA, USA). After thawing, 3T3-L1 cells were resuspended in an α-minimal essential medium (α-MEM; Invitrogen, Carlsbad, CA, USA) supplemented with 10% heat-inactivated fetal bovine serum (F.B.S.; Invitrogen) and 1% antibiotic/antimycotic solution (Invitrogen). The cultures were maintained at 37 °C under an air/5% CO_2_ atmosphere and the medium was changed after 48 h and every 3–4 days thereafter. Upon reaching confluence, the cells were recovered by the addition of 0.25% trypsin/EDTA (Invitrogen) and sub-cultured [17,27,28].

To generate adipocytes overexpressing PGC1-α or deficient in PGC1-α, 1 × 10^6^ cells were seeded in 6-well plates one day before transduction. Adipo-ORF PGC1-α lentivirus and Adipo-sh PGC1-α (Vector builder, Shenandoah, TX, USA) were applied to adipocyte (3T3-L1) cells to establish stably transduced cell lines. The transduction medium consisted of 1 × 10^6^ transducing units (T.U.) of lentiviral particles in 0.5 mL α-MEM growth medium, and this was applied to each well and incubated for 3 h to maximize the contact between the cells and lentiviral particles. The cells were also treated with the transduction medium without the lentiviral particles as described [17,25,26].

### 2.3. Measurement of Fasting Blood Glucose

To evaluate fasting blood glucose levels, mice were fasted for 6 h with free access to water. A glucose-tolerance test was performed at the end of the experiment. Glucose (2 g/kg) was administered to each mouse by intraperitoneal, (i.p.) injection. Blood samples were taken at 0, 30, 60, 90, and 120 min, and glucose was measured using a Contour blood glucose monitoring system (Bayer, Leverkusen, Germany). After these measurements, the blood was collected in capillary tubes and used for insulin measurements. Blood was centrifuged at 2000 rpm for 15 min to separate the plasma. An ultra-Sensitive Mouse Insulin ELISA kit (cat. no. 90080, Crystal Chem., Elk Grove Village, Illinois) was used according to the manufacturer’s instruction to quantify insulin in the plasma.

### 2.4. Western Blot

At the end of the experimental period, the mice were euthanized, organs and tissues collected, snap-frozen in liquid nitrogen, and stored at −80 °C. Frozen mouse adipose and liver tissues were homogenized in lysis buffer containing protease and phosphatase inhibitors. Homogenates were centrifuged and supernatants containing solubilized proteins collected and immunoblotted for PGC-1α, HO-1, OPA1, MFN2, Fis1, UCP1, Sirt1, adiponectin, MnSOD2, Mest, N.O.V., IL-6, Twist1, FGF21, CREG1, PRDM16, AMPK, pAMPK, A.K.T., pAKT, phosphorylation of insulin receptor (I.R.; pIR Tyr^972^, pIRS1 Ser^307^), pSmad 1–5, pSmad2, P38MAPK, pP38MAPK, and β-actin. Immunoreactive bands were quantified using Odyssey Application Software version 3.0.21 [13,29,30].

### 2.5. RT-PCR and R.N.A. Arrays Analysis

Total R.N.A. was obtained from frozen adipose tissue by RNeasy Lipid Tissue (Qiagen, Hilden, Germany), according to the manufacturer’s instructions. The concentration of total R.N.A. was determined by the use of a Take3^®^ plate and a Biotek^®^ Plate Reader (Biotek, Winooski, VT, USA). cDNA was synthesized from 1 μg total R.N.A. and 18 s using the High-Capacity cDNA Reverse Transcription Kit (Applied Biosystems, Foster City, CA, USA), according to the manufacturer’s protocol, and PCR mRNA calculation was adjusted to 18 s as a control as described [13,17,31].

PCR arrays using the RT² Profiler™ PCR Array Mouse Adipogenesis (Qiagen, product no. 330231 and Cat. No. PAMM-049Z) were performed following the manufacturers’ protocols. Gene-expression levels were calculated using the ΔΔCt method after normalization to the housekeeping gene expression (average) and fold change. GeneQuery™ qPCR array kits are qPCR ready in a 96-well plate format, with each well containing one primer set that can specifically recognize and efficiently amplify a target gene’s cDNA. Each GeneQuery™ plate contains eight controls, five target housekeeping genes (β-actin, GAPDH, LDHA, NONO, and PPIH), and genes encoding for pre-adipocyte cell markers, proliferation, differentiation and adipogenesis, lipid metabolism, and obesity. Genes are ordered from the largest increase in expression to the largest decrease in expression in subgroups [32]. In the shortlist, we selected the 10 genes that had the greatest increase in expression and those 10 genes that had the greatest decrease in expression in lean, H.F.D. as well as in adipose-tissue-overexpressing PGC1-α. Again, the color code for the log10 transformed data is shown in the upper left-hand corner of the graph, and H.F. and HF-Transgenic-adipo-PGC-1α were compared with lean. The correlation plot shows the Pearson correlation between different gene expressions in the entire set of data. Details of this method are well described [17,33].

### 2.6. Hematoxylin–Eosin and Masson Trichrome Stain in Liver

The lipid droplets in hepatocytes were assessed on hematoxylin–eosin-stained sections by measuring the diameter (μm) of randomly chosen 100 lipid droplets/groups at a final magnification of 400×. The morphometric analyses were performed by two different observers blinded to the experimental group, using a computer image analysis software (Image Pro Premier 9.1, Media Cybernetics, Rockville, MD, USA).

NAFLD is characterized by liver histopathological features including steatosis, hepatocyte ballooning, inflammation, and fibrosis, as previously described [9,26]. Perivascular hepatic fibrosis evaluation was assessed using Masson trichrome staining and liver fibrosis (blue: collagen fibers; red: hepatocyte cytoplasm). The percentage of perivascular hepatic fibrosis was calculated using a computerized image analyzer (Image Pro Premier 9.1, MediaCybernetics Inc., Rockville, MD, USA) evaluating 20 randomly chosen liver fields per experimental animal [9,26].

### 2.7. Histology, Masson Trichrome Staining, and Immunofluorescence in Adipose Tissues

Adipose and hepatic tissues were collected from all the experimental groups and prepared for morphological analysis. The dissected tissues were fixed in 10% (*vol*/*vol*) neutral-buffered formalin and embedded in paraffin tissue sections were prepared according to standard procedures. Tissue sections of 5 μm thickness were stained with either H&E, or Oil red O (Histoserv, Germantown, MD, USA), or Masson Trichome (Sigma Aldrich, St. Louis, Missouri; to quantify collagen, mainly collagen I and III in blue). Alternatively, sections were processed for immunofluorescence to detect PGC-1α, following the manufacturer’s instructions. The stained slides were analyzed using a microscope, and images captured by a digital camera (Olympus, Tokyo, Japan) and analyzed using ImageJ software. Two hundred cells/sample were included in the analysis of 5 mice for each genotype. The morphological analysis was conducted as previously described [13,17,34].

### 2.8. Measurement of Mitochondrial Oxygen Consumption Rate in Adipocytes

The oxygen consumption rate (O.C.R.) was measured as oxygen consumption per minute (pmols/min). Additionally, extracellular acidification rate (ECAR) was recorded and is a measure of glycolysis; the units are (mpH/min). Extracellular flux analyzer XFp (Seahorse Bioscience, Houston, TX, USA) was used to measure O.C.R. in the cells, which were overexpressed and knockdown for PGC-1α. Adipocytes derived from 3T3-L1 cells were plated at 4 × 10^5^ cells/well into Seahorse 8-well microplate. Oligomycin, FCCP, rotenone, and antimycin were freshly prepared in X.F. assay media. Antimycin A is an inhibitor of ATP synthase, so O.C.R. reduction after antimycin A treatment represents ATP turnover under the specified conditions. FCCP is an uncoupling agent of electron transport and can generate a proton efflux to induce the maximum respiration termed as respiratory capacity or uncoupled respiration [17].

### 2.9. Detection of Nuclear PGC-1α by Immunogold Staining

Mice were sacrificed after the completion of the experiments, and the adipose tissue was collected to perform immunogold staining to determine PGC-1α expression in the nucleus. Glutaraldehyde-fixed samples of mice adipose tissues were cut using an ultramicrotome to obtain ultrathin sections (700 nm) for the post-embedding immunogold analyses, as previously described [35].

### 2.10. Statistics

All data are expressed as means ±standard errors of the mean  S.E.M. The significance of difference in mean values was determined using a one-way ANOVA or repeated-measures two-way ANOVA, followed by a Tukey’s post hoc multiple-comparison test. *p* < 0.05 was considered statistically significant. The *p*-values were denoted as follows: * *p* < 0.05, ** *p* < 0.01, *** *p* < 0.001, **** *p* < 0.0001.

## 3. Results

### 3.1. Transgenic-Adipo-PGC-1α Mice Exhibit Glucose Intolerance

**Figure 1:**
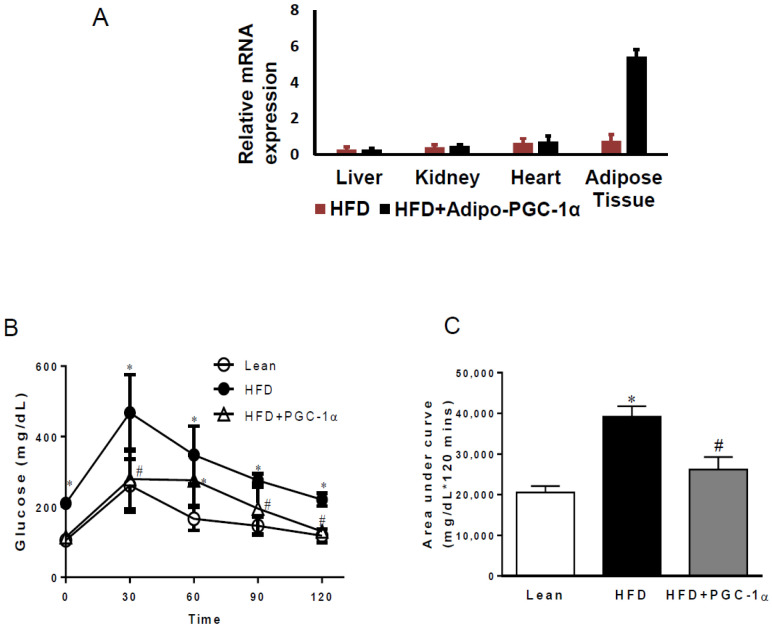
Effects of the adipocyte-specific overexpression of PGC-1α in high-fat-diet (HFD.)-fed mice. (**A**) Tissue expression levels of adipo-PGC1α; (**B**) glucose tolerance test; and (**C**) area under curve; (data are expressed as mean ± SEM (*n* = 5); * *p* < 0.05 versus lean; # *p* < 0.05 vs.HFD.

We first examined the proper expression of adiponectin-driven PGC-1α in different tissues of mice fed a H.F.D. As shown in Figure 1A, adipo-PGC1α was highly expressed (>7-fold) only in the adipose tissue of HFD-fed transgenic mice compared to the control animals, with negligible levels in the liver, kidney, and heart, confirming the adipocyte selectivity of the expression system. Mice expressing adipo-PGC-1α exhibited a decreased impact of H.F.D. on fasting blood glucose levels and an increased tolerance to glucose challenge (*p* < 0.05) compared with H.F.D.-fed mice (Figure 1B,C).

### 3.2. Adipose-Specific Expression of PGC-1α Rescues Mice from HFD.-Induced Adipocyte Hypertrophy, Fibrosis, Liver Steatosis, and Lipid Droplet Size

**Figure 2:**
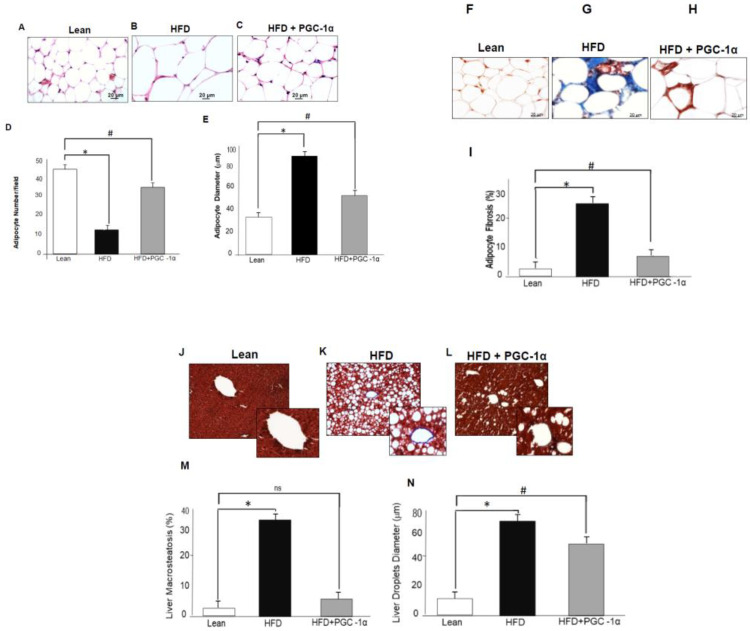
Effects of adipocyte-specific overexpression of PGC-1α in high-fat-diet (HFD)-fed mice compared to lean and untreated HFD mice. Histological analysis of adipose tissue using Hematoxylin–Eosin of (**A**) lean, (**B**) HFD, and (**C**) HFD+ PGC-1α mice; (**D**) Adipocyte number and (**E**) adipocyte diameter. Masson trichrome staining photomicrographs of PGC-1α expression at the adipose tissue level of (**F**) lean, (**G**) HFD, and (**H**) HFD + PGC-1α mice; (**I**) Percentage of adipocyte fibrosis, (**J**–**M**) liver macrosteatosis, and (**N**) liver lipid droplet diameter. Bar 20 μm. Data are expressed as mean ± S.E.M. (*n* = 5); * *p* < 0.05 versus lean; # *p* < 0.05 vs. HFD.

We analyzed adipocyte diameter and fibrosis in the adipose tissue of lean, HFD-fed and HFD-fed Transgenic-adipo-PGC-1α mice. As shown in Figure 2, obese mice (HFD-fed) had enlarged adipocytes (*p* < 0.05), but in reduced numbers, compared to the lean mice (Figure 2A–E). The overexpression of PGC-1α attenuated adipocyte hypertrophy and restored adipocyte numbers (Figure 2, *p* < 0.05). Adipocyte hypertrophy and fibrosis were also reversed in Transgenic-adipo-PGC-1α mice (*p* < 0.05 vs. HFD-fed mice, Figure 2F–I). The increase in brown adipocytes associated with increased PGC-1α expression was accompanied by reduced liver steatosis and lipid droplet size in transgenic-PGC-1α mice (Figure 2J–N).

### 3.3. Nuclear Localization of PGC-1α in Adipocytes

**Figure 3:**
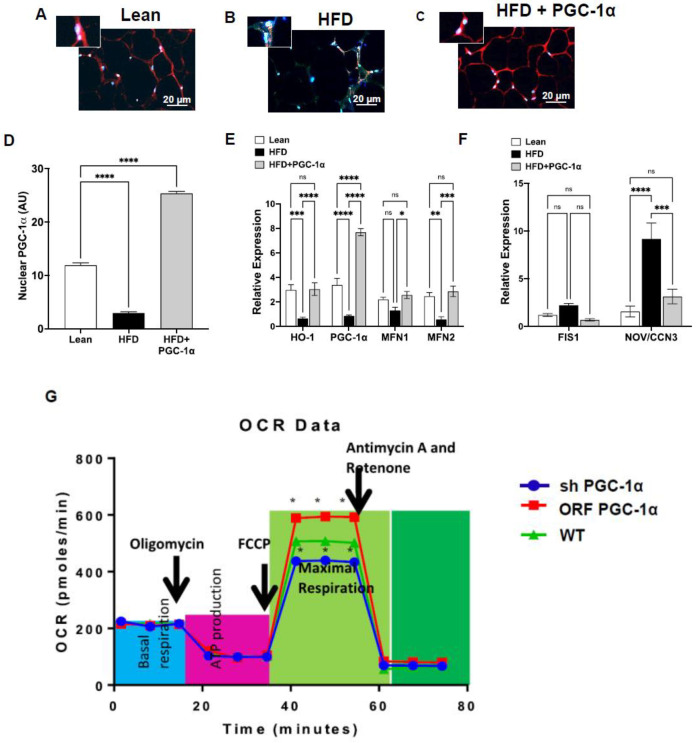
Effects of the adipocyte-specific overexpression of PGC-1α in high-fat-diet (H.F.D.)-fed mice compared to lean and untreated HFD mice. Iimmunofluorescence photomicrographs of PGC-1α expression (red staining) at the adipose tissue level of (**A**) lean, (**B**) H.F.D., and (**C**) HFD + PGC-1α mice. Graph (**D**) summarizes the immune morphometrical measurement of the nuclear localization of PGC-1α (A.U.). Quantitative gene expression analysis of (**E**) PGC-1α, HO-1, Mfn1, and Mfn2; (**F**) Fis1 and NOV/CCN3 in adipose tissue of Transgenic-adipo-PGC-1α mice fed with a high-fat diet. Data are expressed as mean ± S.E.M. (**n** = 5); **** *p* < 0.05 versus lean; *** *p* < 0.05 versus HFD,* *p* < 0.05 versus HFD. ** *p* < 0.05 versus lean (**G**) Oxygen consumption rates (OCR) in PGC-1α knockdown (shPGC1α), overexpression (ORF PGC-1α), and control cultured adipocytes, *n* = 3. **** *p* < 0.005, versus control. The maximal respiration capacity represents the sum of all physiological mitochondrial oxygen consumption.

The transcriptional activity of PGC-1α requires its localization in the nucleus. Figure 3 shows the subcellular localization of PGC-1α (red fluorescence; nuclei shown in blue by DAPI staining) in the adipocytes from the three groups of animals. HFD mice showed a reduced (*p* < 0.05) nuclear localization of PGC-1α compared to lean mice. Importantly, this was not only reversed, but even increased, in the Transgenic-Adipo-PGC-1α mice fed an HFD (*p* < 0.05) (Figure 3A–D), when compared to the lean animals.

### 3.4. mRNA Levels of PGC-1α, HO-1, Mitochondrial Genes, and NOV/CCN3 in the Adipose Tissue of Transgenic-Adipo-PGC-1α Mice

The mRNA levels of HO-1, PGC-1α, and the mitochondrial fusion-associated proteins Mfn1 and Mfn2 were decreased in the H.F.D. group as compared to the lean group (*p* < 0.05); the effect of a H.F.D. was reversed in the adipose tissues from Transgenic-adipo-PGC-1α animals (Figure 3E). Conversely, RT-PCR results demonstrated increased mRNA levels of mito-fission-related Fis1 and NOV/CCN3 in the adipose tissue of the control H.F.D.-fed mice (Figure 3F), an effect of obesity that was prevented in the adipose tissue of the Transgenic-adipo-PGC-1α mice fed a H.F.D. Moreover, the mRNA levels of TNF-α, IL1β, and CCL2 in the H.F.D. group were increased (*p* < 0.05) compared with those of the Lean group (not shown). The expression of these genes was reduced (*p* < 0.05) in the adipose tissues of Transgenic-adipo- PGC-1α mice on H.F.D. 

### 3.5. PGC-1α Mediated Regulation of Mitochondrial Function in Cultured Adipocytes

To analyze the role of PGC-1α in adipocytes in the context of mitochondrial bioenergetics, we measured mitochondrial respiration in control, knockdown (sh PGC-1α), and overexpressed PGC-1α (ORF PGC-α) adipocyte cells. Real-time oxygen consumption rates (O.C.R.s) in adipocytes showed that basal respiration, representing the sum of all physiological mitochondrial oxygen consumption, was decreased in the mitochondria of PGC-1α deficient cells, indicating a lower respiratory function when compared with that of the control cells, which was rescued after the overexpression of PGC-1α (Figure 3G). Oligomycin decreased basal respiration, reflective of the fraction of oxygen consumption used to generate ATP. In the presence of carbonyl cyanide-*p*-trifluoromethoxy-phenylhydrazone (FCCP), PGC-1α-deficient cells had a lower O.C.R., which was rescued by the overexpression of PGC-1α, indicating a lower overall mitochondrial activity. The extent of non-mitochondrial oxygen-consuming processes was estimated by inhibiting the respiratory chain with rotenone and antimycin A; there was no change in PGC-1α-ablated-cell mitochondria, which were rescued after the overexpression of PGC-1α. ATP turnover was decreased in PGC-1α-ablated cells. The maximum respiration was also lower in PGC-1α-deficient cells, which was rescued after the overexpression of PGC-1α (*p* < 0.05) (Figure 3G).

### 3.6. Expression Levels of Mitochondrial Biogenesis and Fusion Genes, UCP1, and Antioxidant-Associated Proteins in Transgenic-Adipo-PGC-1α Mice

**Figure 4:**
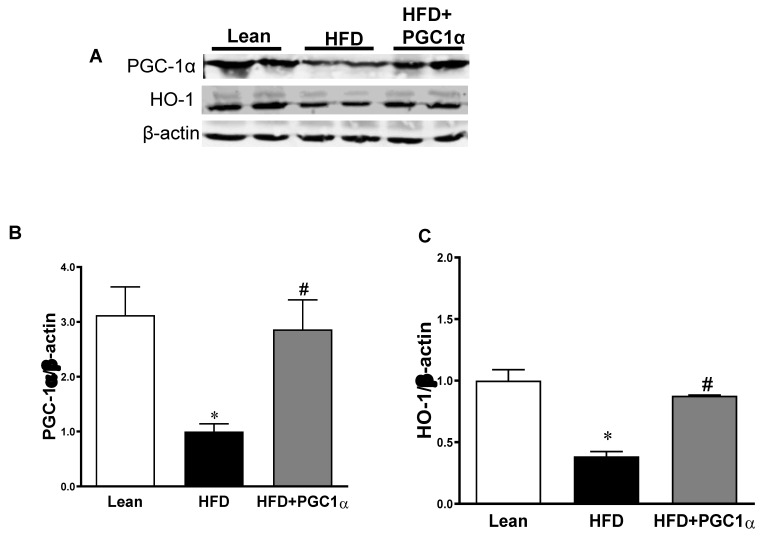

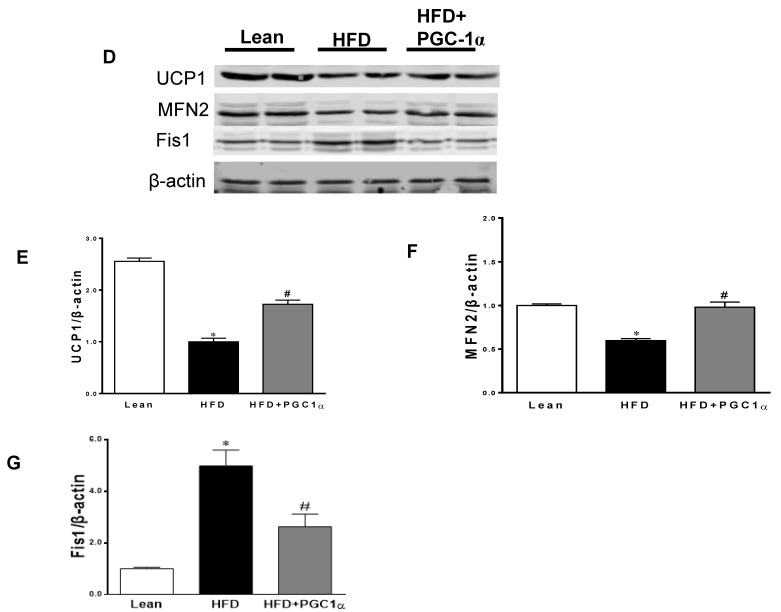

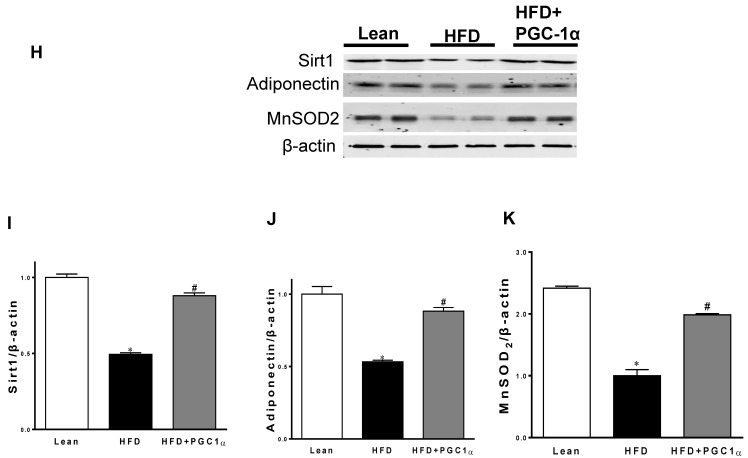
Effects of the Transgenic-adipo-PGC-1α treatment on the key proteins involved in mitochondrial fission and biogenesis and adipocyte browning. Representative Western blot analysis of (**A**) PGC-1α and HO-1 (**B**,**C**), (**D**–**G**) MFN2, Fis1, and UCP1, (**H**–**K**) Sirt1, Adiponectin and MnSOD2 with their corresponding β-actin in the adipose tissue of lean, HFD and Transgenic-adipo-PGC-1α mice. Data are expressed as mean ± S.E.M. (*n* = 5); * *p* < 0.05 versus lean; # *p* < 0.05 versus HFD.

As shown in Figure 4, the protein levels of PGC-1α and HO-1 were reduced in the adipose tissue of H.F.D.-fed mice as compared to those in the lean animals (*p* < 0.05), but were increased in Transgenic-adipo-PGC-1α mice (*p* < 0.05) (Figure 4A–C). In addition, the uncoupling protein 1 (UCP1) was increased (*p* < 0.05) in the adipose tissues of mice treated with Lenti-adipo-PGC-1α (Figure 4D,E). We examined the effect of a H.F.D. on mitochondrial fusion and fission genes and found that Mitofusin2 (MFN2) and OPA1 were decreased in the H.F.D. group, whereas Fis2 was increased (*p* < 0.05). These effects were reversed in Transgenic-adipo-PGC-1α mice (Figure 4D,F,G). Furthermore, Transgenic-adipo-PGC-1α mice expressed higher (*p* < 0.05) levels of Sirt1, adiponectin, and MnSOD2 proteins compared with the H.F.D.-fed mice (Figure 4H–K).

### 3.7. Expression Levels of Inflammatory Mediators, Insulin Signaling Components, and AMPK in the Adipose Tissue of Transgenic-Adipo-PGC-1α Mice

**Figure 5:**
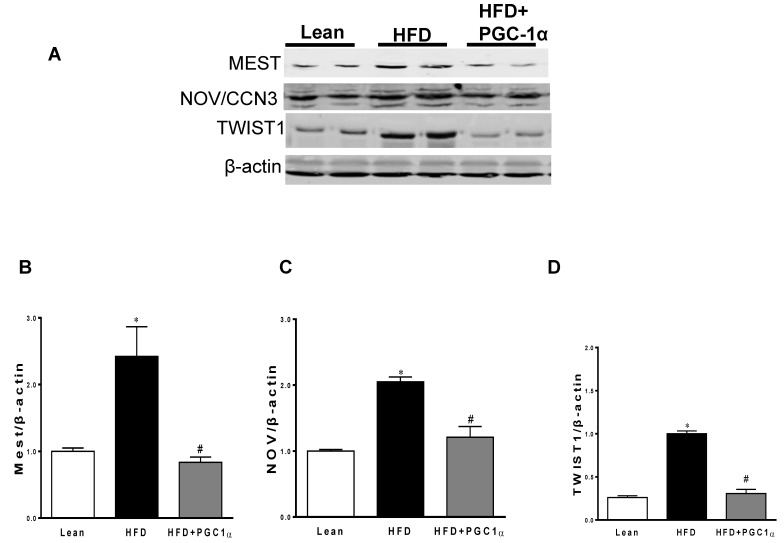

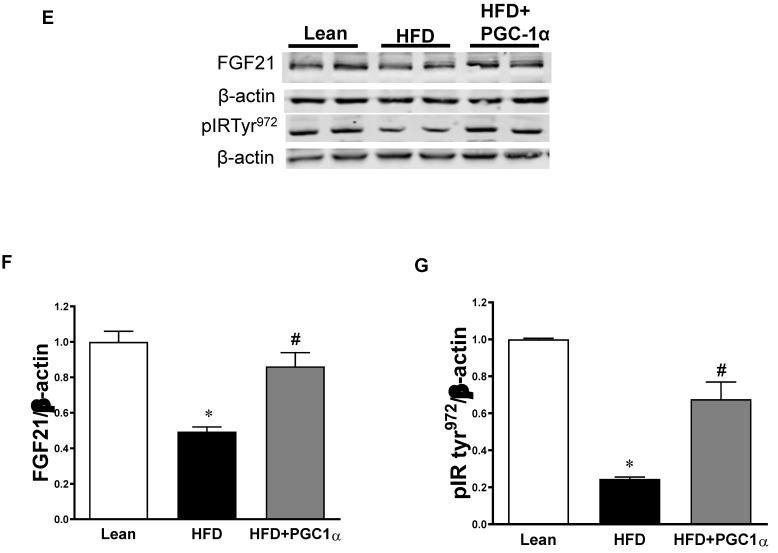

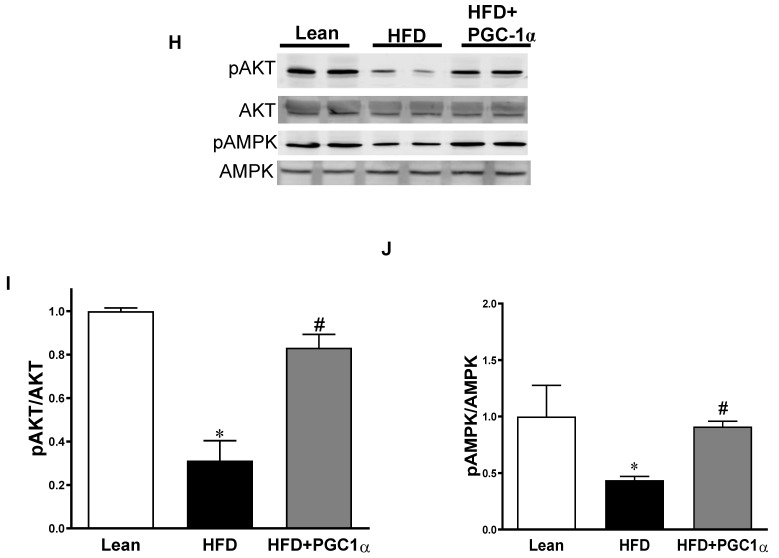
Expression of inflammatory mediators and insulin signaling in the adipose tissue of lean, high-fat-diet (H.F.D.)-fed, and Transgenic-adipo-PGC-1α mice. Representative Western blot analysis of (**A**–**D**) MEST, NOV/CCN3 and TWIST, (**E**–**G**) FGF21 and phosphorylated insulin receptor tyrosine 972 (pIRTyr972), (**H**–**J**) pAKT and pAMPK with their corresponding A.K.T. and AMPK in the adipose tissue of lean, H.F.D., and Transgenic-adipo-PGC-1α mice. Data are expressed as mean ± S.E.M. (*n* = 5); * *p* < 0.05 vs. lean; # *p* < 0.05 vs. H.F.D.

The protein expression of the mesoderm-specific transcript (Mest) in the adipose tissue of the HFD-fed mice was increased as compared to that of the lean group (Figure 5A,B). In the HFD-fed Transgenic-adipo-PGC-1α mice, MEST expression was reduced (*p* < 0.05) compared with that of the control H.F.D.-fed mice. As shown in Figure 5A,C,D, N.O.V. and Twist1 protein expressions in the adipose tissue of Transgenic-adipo-PGC-1α mice were reduced (*p* < 0.05) as compared to those of the H.F.D.-fed mice. In addition, the adipose tissue of H.F.D. mice exhibited lower levels of FGF21, pIR tyr^972^, pAKT, and pAMPK than those in the lean mice. The expression of all these proteins was normalized in the Transgenic-adipo-PGC-1α animals (Figure 5E–J).

### 3.8. Identification of Changes in the Correlation Coefficients of Gene Expression by RNA Array Analyses

**Figure 6:**
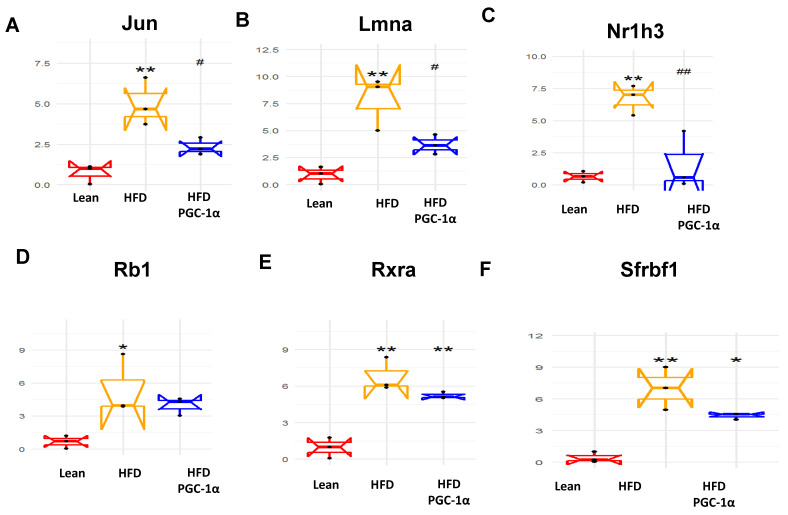
The mRNA expression of (**A**) Jun proto-oncogene (Jun), (**B**) lamin A (Lmna), (**C**) nuclear receptor subfamily 1 group H member 3 (Nr1h3), (**D**) R.B. transcriptional corepressor 1 (Rb1), (**E**) retinoid X receptor alpha (Rxra), and (**F**) secreted frizzled-related protein 1 (Sfrbf1) in the adipose tissue of lean, high-fat-diet (H.F.D.)-fed and Transgenic-adipo-PGC-1α mice. Data are expressed as mean ± S.E.M. (*n* = 5); * *p* < 0.05, ** *p* < 0.005 vs. lean; # *p* < 0.05, ## *p* < 0.005 vs. HFD.

We previously examined the effect of thymoquinone on obesity-induced inflammation, insulin resistance, and the metabolic browning of adipose tissues [36]. We also wanted to study the effect of PGC-1α expression during adipogenesis, and we examined 88 genes that are exclusively expressed in adipocytes before and during adipogenesis [32]. As shown in Figure 6A–F, the mRNA levels of Jun, Lmna, Nr1h3, Rb1, Rxra, and Sfrbf1 were upregulated in the adipose tissues of HFD mice compared to their corresponding levels in the adipose tissue of the lean mice. In contrast, when compared to the HFD-fed mice, the levels of all these genes were reduced, in some cases almost to lean-mice levels in the adipose tissue of the transgenic-adipo-PGC-1α HFD-fed mice (Figure 6A–F).

### 3.9. Upregulated Genes in the Adipose Tissues of Transgenic-Adipo-PGC1 Mice

**Figure 7:**
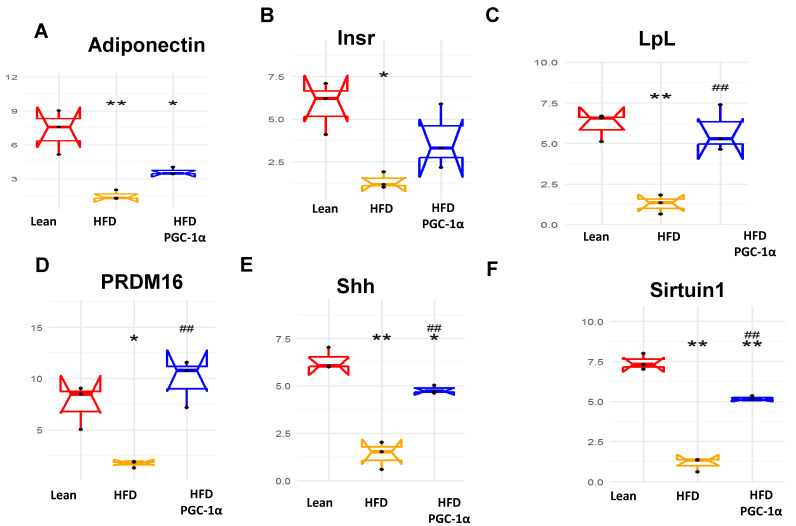

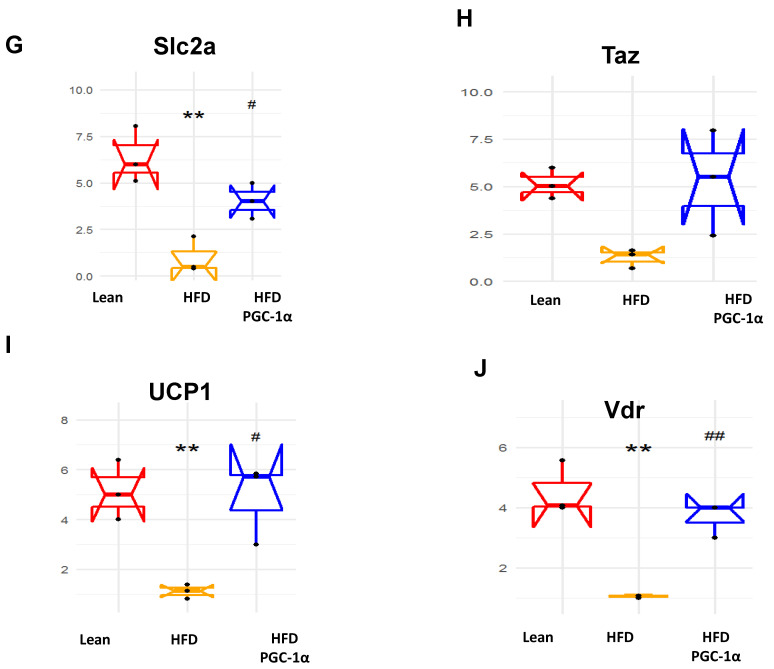
The mRNA expression of (**A**) adiponectin, (**B**) insulin receptor precursor (Insr), (**C**) lipoprotein lipase (Lpl), (**D**) Prdm16, (**E**) Sonic hedgehog (Shh), (**F**) silent mating-type information regulation 2 homolog (Situin1), (**G**) solute carrier family 2 member 4 (Slc2), (**H**) Tafazzin (Taz), (**I**) Uncoupling protein 1 (UCP1), and (**J**) vitamin D receptor (Vdr) in the adipose tissue of lean, high-fat-diet (H.F.D.)-fed and Transgenic-adipo-PGC-1α mice. Data are expressed as mean ± S.E.M. (*n* = 5); * *p* < 0.05, ** *p* < 0.005 vs. lean; # *p* < 0.05, ## *p* < 0.005 vs. HFD.

Figure 7 shows the effect of a H.F.D. on the expression levels of adiponectin, Insr, LpL, PRMD16, Shh, Sirtuin 1, Slc2a, Taz, UCP1, and Vdr (Figure 7A–J). All these genes were downregulated (* *p* < 0.05, ** *p* < 0.005) in the adipose tissues of H.F.D. mice, as compared to their corresponding levels in the adipose tissues of the lean mice. As expected, these genes were upregulated (# *p* < 0.05, ## *p* < 0.005) in the adipose tissues of transgenic-adipo-PGC-1α HFD mice as compared to their levels in the H.F.D. mice. Specifically, PRMD16, UCP1, Sirtuin1 and adiponectin are known to be positively regulated by PGC-1α and participate in the ‘browning’ of fat tissue. The robust increase in UCP1 and PRMD16 observed in the adipose tissue of transgenic-adipo-PGC-1α H.F.D. mice is of particular significance since this suggests increased energy consumption and mitochondrial respiration (Figure 7A–J).

### 3.10. Effect of HO-1 Inhibition on the Phosphorylation of Insulin Receptor and Markers of Brown-like Fat in Transgenic-Adipo-PGC-1α Mice

**Figure 8:**
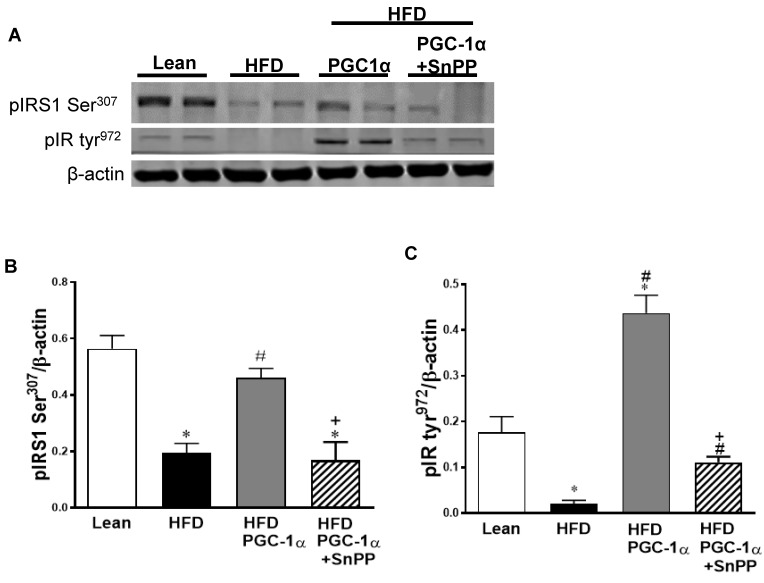

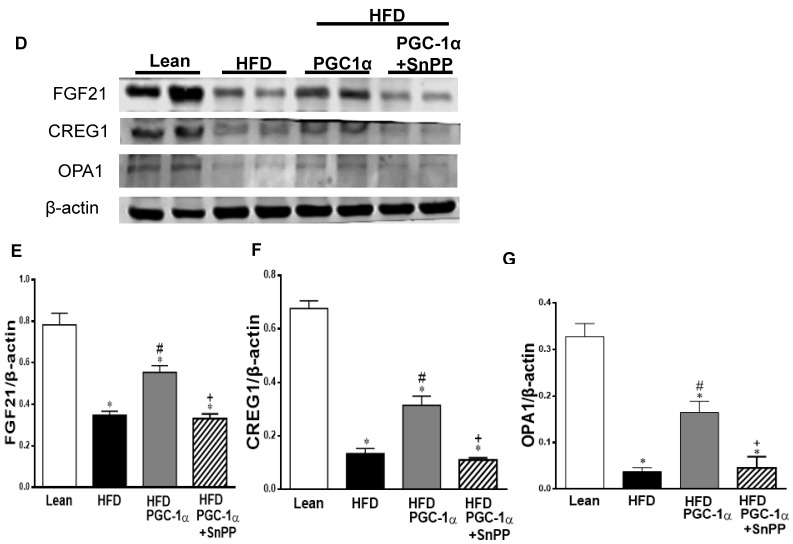
Effects of SnPP treatment on insulin-receptor phosphorylation and mitochondrial fission in the adipose tissue of Transgenic-adipo-PGC-1α mice fed with high-fat diet (H.F.D.). Representative Western blots of (**A**) phosphorylated insulin-receptor substrate 1 serine^307^ (pIRS1 ser^307^) and phosphorylated insulin-receptor tyrosine^972^ (pIR Tyr^972^) with the corresponding quantitation to β-actin (**B**,**C**). (**D**) Representative Western blots of FGF21, CREG1, and OPA1 with the corresponding quantitation to β-actin (**E**–**G**). Data are expressed as mean ± S.E.M. (*n* = 5); * *p* < 0.05 versus lean; # *p* < 0.05 versus H.F.D.; + *p* < 0.05 vs. PGC-1α.

As shown in Figure 8A–C, the adipose tissue of the H.F.D. mice showed a lower level of phosphorylation of the insulin-receptor substrate 1 Ser^307^ (pIRS1 Ser^307^) and the receptor itself (pIR tyr^972^) compared to that of the lean group (*p* < 0.05). In the transgenic-adipo-PGC-1α mice fed an H.F.D, however, the phosphorylation levels of these two proteins were restored.

The inhibition of H.O. activity by SnPP in the Transgenic-adipo-PGC-1α mice fed with an HFDreversed the increase in the phosphorylation of pIRS1 Ser^307^ and pIR tyr^972^ (*p* < 0.05).

FGF21 and CREG1 are key transcriptional regulators in brown and beige adipocyte formation, and optic atrophy-1 (OPA1) controls mitochondrial fusion processes. Figure 8D–G shows reduced levels of FGF21, CREG1, and OPA1 in the adipose tissue of HFD-fed mice as compared to those of the lean animals, an effect that was normalized in transgenic-adipo-PGC-1α H.F.D.-fed mice. Once again, SnPP downregulated FGF21, CREG1, and OPA1 protein expression in the Transgenic-adipo-PGC1α mice.

### 3.11. Effect of SnPP on the Expression of the PGC-1α Target

**Figure 9:**
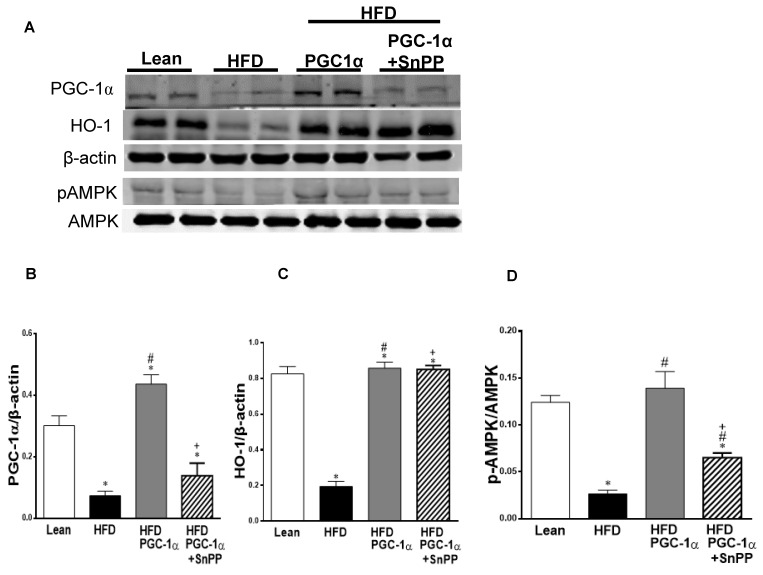

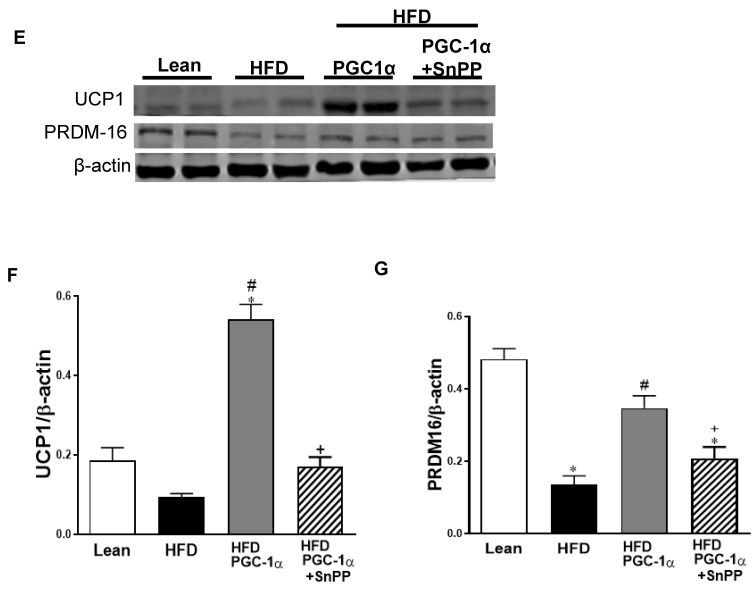

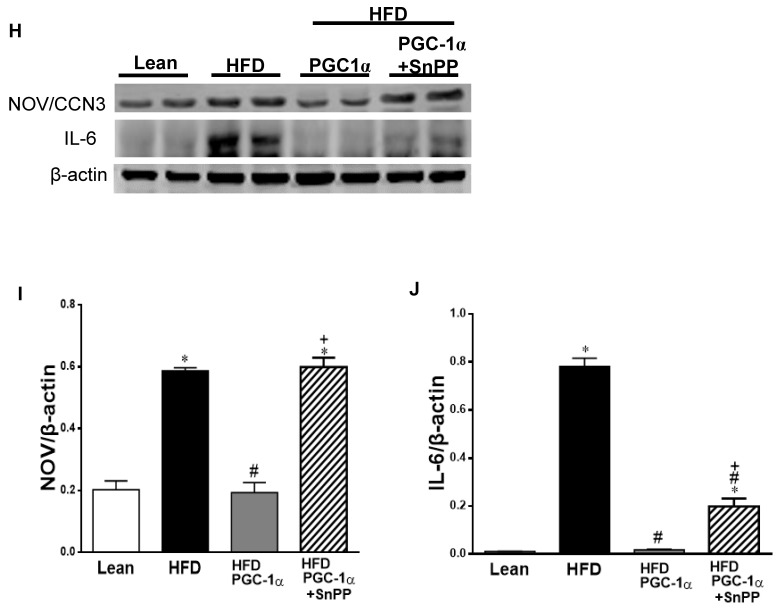

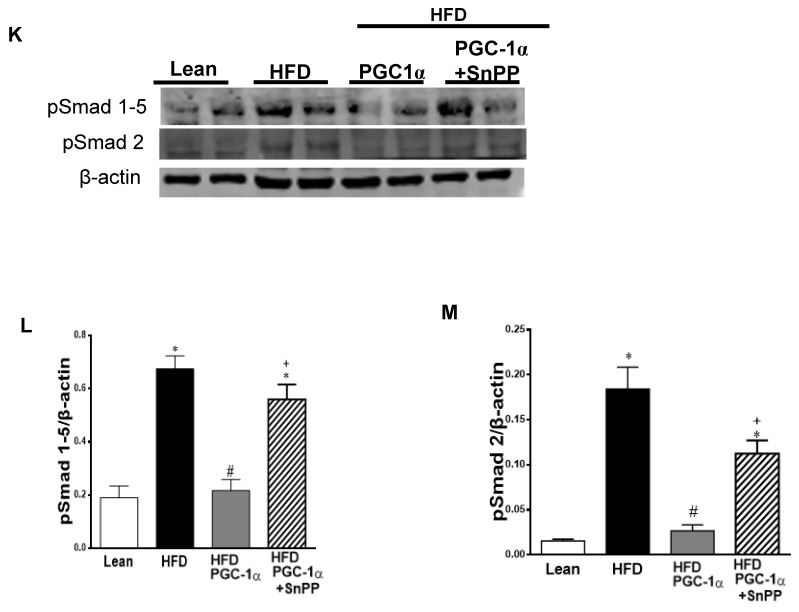
Effects of SnPP treatment on browning markers and AMPK phosphorylation in the adipose tissue of Transgenic-adipo-PGC-1α mice fed with a high-fat diet. Representative Western blots of (**A**) PGC-1α, HO-1, pAMPK, and AMPK with the corresponding quantitation to β-actin (**B**–**D**). (**E**–**G**) UCP1 and PRDM16. Data are expressed as mean ± S.E.M. (*n* = 5); * *p* < 0.05 vs. lean; # *p* < 0.05 versus H.F.D.; + *p* < 0.05 vs. PGC-1α. Effects of SnPP treatment on inflammatory mediators and transforming growth factor β (TGF-β) signaling (pSmad 1–5 and pSmad 2) in the adipose tissue of Transgenic-adipo-PGC-1α mice fed with a high-fat diet. Representative Western blots of (**H**–**J**) NOV/CCN3 (**H**) and IL-6 (**J**) with the corresponding quantitation to β-actin; Representative Western blots of pSmad 1–5 and pSmad2 with the corresponding quantitation to β-actin (9; **K**–**M**); and phosphorylation of pP38 MAPK (9N) with the corresponding quantitation to pP38 MAPK. Data are expressed as mean ± S.E.M. (*n* = 5); * *p* < 0.05 versus lean; # *p* < 0.05 versus H.F.D.; + *p* < 0.05 versus PGC-1α.

In adipose tissues, AMPK phosphorylation plays a role in insulin-receptor phosphorylation. As shown in Figure 9A,D the levels of pAMPK were reduced in mice fed with a H.F.D. compared to the levels in the lean mice. In contrast, pAMPK was increased (*p* < 0.05) in the Transgenic-adipo-PGC-1α mice and prevented by SnPP. The same pattern was observed in the expression of UCP1 and PRDM16 (Figure 9E–G). These data suggest that adipose tissue PGC-1α mediates beneficial effects on the insulin signaling pathway in an HO-1-dependent manner. (Figure 9A–G).

The effect of SnPP on inflammation and TFG-β signaling is shown in Figure 9H–N. We observed that SnPP increased the expression of NOV/CCN3 and IL-6 in adipose tissues, as compared to the lean mice fed with a normal chow diet.

The protein levels of NOV/CCN3 and IL-6 in the adipose tissue of Transgenic-adipo-GC-1α mice fed with an HFD were reduced when compared to the mice fed with a HFD (*p* < 0.05). Interestingly, the levels of these proteins were (*p* < 0.05) increased when the transgenic-adipo-PGC-1α mice were treated with SnPP (Figure 9H–J).

The phosphorylation of Smad1–5 and Smad2, as well as that of *p* P38MAPK in adipose tissues were significantly elevated in the H.F.D. group as compared to the lean group (Figure 9K–N), while these increases were normalized in the PGC-1α-transduced mice (*p* < 0.05). SnPP blunted the inhibitory effects of PGC-1α on pSmad 1–5, pSmad 2 and p38MAPK. These data suggest that PGC-1α suppresses the activation of the TGF-β/Smad signaling pathways, an effect that was clearly dependent on HO-1 (Figure 9K–N).

## 4. Discussion

We provided evidence demonstrating that the adipocyte-specific overexpression of PGC-1α ameliorates metabolic dysfunction in mice fed with an HFD, as evidenced by improved insulin sensitivity. In addition, an examination of gene profile expression in adipose tissues revealed that these effects are related to PGC-1α promoting the reprogramming of white fat into brown adipocytes, with improved oxygen consumption and increased levels of adiponectin. The impact of the adipocyte-selective expression of PGC-1α was also evident on distal tissues. Transgenic-adipo-PGC-1α mice displayed reduced adipocyte hypertrophy, associated with a reduction in molecules within the TGF-β signaling pathway, and of the inflammatory markers nephroblastoma-overexpressed/cellular communication network factor 3 (NOV/CCN3), Twist1, and IL-6. In contrast, mitochondrial biogenesis, fission, and expression of genes linked to beige adipocytes were upregulated in the Transgenic-adipo-PGC-1α mice. The improvement in metabolic function by selective PGC-1 α expression was HO-1-dependent, since SnPP abrogated the positive effects of PGC-1α on both adipocyte browning and inflammation, as well as attenuating the improvement in cardiometabolic function.

PGC-1α is one of the key regulators of mitochondrial biogenesis and cellular energy metabolism [37]. PGC-1α has been shown to regulate HO-1 expression and is cardioprotective in epicardial fat, attenuating cardiovascular risk [31,32]. The central role of PGC-1α in adipose tissue regarding energy homeostasis has been established in mice with the adipocyte-specific deletion of PGC-1α, which results in insulin resistance and increased lipidemia when animals are fed with an HFD [38]. It is widely recognized that a close relationship exists between PGC-1α activity, insulin sensitivity, and the prevention of type 2 diabetes, most likely due to the essential role of PGC-1α in mitochondrial biogenesis and glucose/fatty acid metabolism [39]. PGC-1α is highly expressed in tissues with a high-energy demand, such as the brain, heart, liver, and kidney [37], and its induction, along with sirtuin 1 (Sirt1) and AMPK, play a key regulatory role in mitochondrial biogenesis upon energy stress [40]. We showed that PGC-1α expression in the adipose tissue of mice fed with an HFD resulted in a decrease in mitochondrial fission genes, such as mitochondrial fission 1 protein (FIS1), and an increase in mitochondrial fusion-associated genes, such as mitofusion 2 (MFN2). Adipocyte dysfunction is manifested by the secretion of pro-inflammatory adipocytokines, such as TNFα, IL1β, and IL6, which also adversely affect vascular tone [7]. Our data show an elevation in the pro-inflammatory and pro-fibrotic protein N.O.V., which was reduced in adipocytes overexpressing PGC-1α. We have shown in previous work in obese mice that the expression of N.O.V. negatively correlates with HO-1 and PGC-1α levels and with mitochondrial function [26,28,36]. Not surprisingly, N.O.V. plasma levels positively correlate with obesity and metabolic syndrome [41,42], and N.O.V. is highly expressed in the epicardial fat of obese patients [32]. Obesity-induced inflammation has been reversed by APO A-1 mimetic-like proteins via HO-1 upregulation [43] and accompanies the upregulation of the Wnt canonical signaling cascade [43,44,45]. We show the important relationship of PGC1α and HO-1 in reversing the effects of obesity on cardiometabolic dysfunction.

The transforming growth factor (TGF-β) family transmits signals via serine/threonine kinase receptors and transcription factors called Smads, and is a central regulator of fibrogenesis in a number of tissues, including the liver [46]. A recent study indicates that TGF-β negatively regulates the presumptive beige progenitor cells in white fat, with the potential to switch to beige adipocytes [47]. Thus, interference with TGF-β/Smad signaling via the manipulation of PGC-1α provides a promising approach for the development of therapeutic interventions for obesity and the metabolic syndrome [48]. It is reported that TGF-β inhibition in Smad^3−/−^ mice increases PGC-1α expression, which mediates the induction of mitochondrial biogenesis and uncoupling protein 1 of brown adipocyte (UCP1) expression [49]. The data presented in this paper further suggest that adipose tissues with an increased expression of PGC-1α and concomitant HO-1 induction suppresses TGF-β signaling and the inflammatory mediators that prevent the formation of beige adipocytes, providing a link between PGC-1α and TGF-β in an HO-1-dependent manner.

Increased adiposity and adipocyte inflammation impair insulin signaling and contribute to the development of insulin resistance and metabolic dysfunction [50], which are characterized by the inhibition of the antioxidant genes PGC-1α and HO-1 [31]. PGC-1α acts upstream of HO-1 and is required for HO-1 expression in cultured adipocytes; in vivo, knocking down PGC-1α abolishes the beneficial effect of HO-1 on adiposity, cardiac function, insulin signaling, and mitochondrial function and biogenesis [13,27,28,29]. Increased adiposity and insulin resistance also impair liver function, leading to NAFLD (nonalcoholic fatty liver disease) that can progress to NASH (nonalcoholic steatohepatitis). The development of NAFLD and/or NASH is closely related to overnutrition and obesity, as well as to inflammation [9,51,52], increased R.O.S. production, and mitochondrial dysfunction [53]. Several models of NAFLD highlight the importance of the anti-oxidative system and mitochondrial genes in the maintenance of liver function and the prevention of the progression of fatty liver to NAFLD [26,36,54,55,56].

We also studied the effect of PGC-1α expression on the process of adipogenesis, and we examined 88 genes that are exclusively expressed in adipocytes before and during adipogenesis [32]. The mRNA levels of Jun, Lmna, Nr1h3, Rb1, Rxra, Sfrbf1 were upregulated in the adipose tissues of HFD mice, compared to their corresponding levels in the adipose tissue of the lean mice. In contrast, when compared to the HFD-fed mice, all these genes were reduced, in some cases almost to lean-mice levels in the adipose tissue of the transgenic-adipo-PGC-1α HFD-fed mice. We showed the effect of HFD on the expression levels of adiponectin, Insr, LpL, PRMD16, Shh, Sirtuin 1, Slc2a, Taz, UCP1, and Vdr. All these genes were downregulated in the epididymal adipose tissues of HFD mice, as compared to their corresponding levels in the adipose tissues of the lean mice. These genes were upregulated in the adipose tissues of the transgenic-adipo-PGC-1α HFD mice as compared to their levels in the HFD mice. Specifically, PRDM 16, UCP1, Sirtuin1, and adiponectin are known to be positively regulated by PGC-1α and participate in the ‘browning’ of fat tissue. The robust increase in UCP1 and PRDM16 observed in adipose tissues from transgenic-adipo-PGC-1α HFD mice is significant, since this suggests increased energy consumption and mitochondrial respiration.

We showed that the pharmacological induction of PGC-1α and HO-1 as well as adipocyte-specific HO-1 overexpression ameliorates NAFLD and NASH in obese mice [9,26,57,58,59]. In the present study, the specific PGC-1α overexpression in adipocytes was sufficient to ameliorate liver steatosis and reduce the NAFLD activity score (N.A.S.) in obese mice, suggesting a paracrine or endocrine influence of adipocyte PGC-1α in improving liver function and morphology.

Our results support the hypothesis that PGC-1α induction in adipocytes leads to HO-1 expression to satisfy its requirement for increased levels of HO-1 activity and to regulate critical aspects of insulin signaling, mitochondrial integrity, anti-inflammatory effects, and adipose tissue browning. The present results indicate and confirm previous reports that the recruitment of HO-1 is essential to mediate the beneficial effects of PGC-1α on the improvement of mitochondrial function, adipocyte browning, vascular tone, and reduced inflammation [60,61,62].

## 5. Conclusions

**Figure 10:**
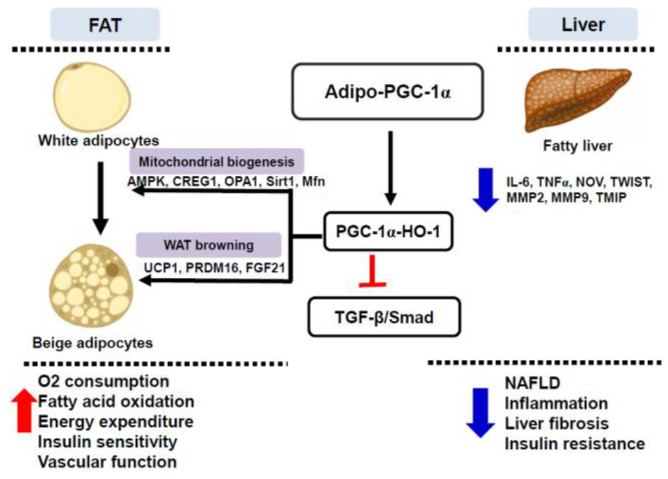
Schematic depiction of the postulated hypothesis showing that adipocyte-specific PGC-1α expression coordinates HO-1 to induce the conversion of white adipocytes to the beige phenotype, and improves mitochondrial biogenesis/fusion, energy expenditure, and insulin sensitivity as well as vascular function. Adipo-PGC-1α inhibits TGF-β/Smad signaling (pSmad 1–5, Smad 2 and P38MAPK) and inflammatory adipokines (NOV/CCN3 and IL-6). Selective expression of adipocyte PGC-1α provides a genetic approach for obesity, fatty liver, and associated metabolic syndrome management.

The adipocyte-specific overexpression of PGC-1α promotes the conversion of white adipocytes to a beige phenotype, with the subsequent reduction in inflammation and improvement in insulin sensitivity. This can be attributed, in part, to the inhibition of adipocyte-derived N.O.V., but also of the TGF-β signaling pathway. The improvement in mitochondrial function and insulin signaling also suggests the involvement of autophagy as one of the mechanisms potentially upregulated in transgenic-adipo-PGC-1α mice that can contribute to the reprogramming of white adipocytes into brown cells through the stimulation of UCP1 and PRDM16. Our findings unveil the manipulated expression of adipocyte PGC-1α as a novel strategy with potential therapeutic impact to improve obesity-linked cardiometabolic diseases (Figure 10).

## Data Availability

The data are contained within the article.
